# FcγRIIIA -activating antibodies in dengue virus infection reveals a distinct transient cross-reactive profile

**DOI:** 10.3389/fimmu.2025.1662138

**Published:** 2025-09-29

**Authors:** Claudio Soto-Garita, Tatiana Murillo, Hartmut Hengel, Eugenia Corrales-Aguilar

**Affiliations:** ^1^ Virology Section, Research Centre for Tropical Diseases and Faculty of Microbiology, University of Costa Rica, San José, Costa Rica; ^2^ Institute of Virology, University Medical Center, Faculty of Medicine, University of Freiburg, Freiburg, Germany

**Keywords:** DENV, humoral response, antibody-dependent enhancement (ADE), FcγRIIIA, CD16A

## Abstract

Dengue viruses belong to the genus Flavivirus and consist of a serocomplex of four serotypes (DENV-1, DENV-2, DENV-3, and DENV-4). As arthropod-borne viruses (arboviruses), their transmission is mediated primarily by the vector Aedes aegypti. Antiviral immune response is one of the most crucial factors influencing the progression from uncomplicated to severe dengue virus (DENV) infection. Two types of antibody responses are elicited during a DENV infection: one specific to the infecting serotype (serotype-specific or homotypic response) and another that cross-reacts with other serotypes (cross-reactive or heterotypic response). Both responses play roles in the protection against and in the induction of immunopathogenesis of DENV disease. In the case of the humoral immune response, the balance between protective and pathogenic effects mediated by antibodies (antibody-dependent enhancement, ADE) is highly dynamic and influenced by multiple factors. Although many downstream effector mechanisms depend on antibody recognition by Fc-gamma receptors (FcγRs) present on immune effector cells, this interaction is traditionally not considered when evaluating antibody properties. Specifically, FcγRIIIA has been implicated in both protection and immunopathogenesis of virus infection. To assess its role within the humoral immune response to DENV, we took advantage of FcγRIIIA-CD3ζ reporter cells and tested receptor activation by polyclonal sera from individuals with past and acute DENV infections. In addition, the neutralizing capacity and the potential enhancement of infection were analyzed. The FcγRIIIA activation assay revealed a humoral profile distinct from neutralization and immunopotentiation, primarily mediated by cross-reactive antibodies. Notably, this profile increases during the post-acute period but disappears within two years after infection. Because these two types of antibodies are found during both the cross-protective and disease-enhancing (immunopotentiation) phases, their exact function in each situation is still not clearly understood. The results of this study provide a valuable measurement of the effector function of anti-DENV antibodies, contributing to the understanding of their role in both protective and disease enhancing courses of DENV infection.

## 1 Introduction

Dengue virus (DENV) is an arthropod-borne virus (arbovirus) transmitted by *Aedes aegypti* and *Aedes albopictus*, being the former the most important vector. DENV is assigned to the family *Flaviviridae* and the genus *Orthoflavivirus* and poses a major global health burden in tropical and subtropical regions. The World Health Organization (WHO) estimates that between 100 to 400 million infections occur yearly and that half the world population is at risk of infection (1). Costa Rica is considered a hyperendemic country for dengue, with co-circulation of all four DENV serotypes and recurring outbreaks that continue to pose major public health concerns (2). DENV infection is characterized by an incubation period of 4 to 10 days after the mosquito bites and produces a spectrum of clinical manifestations. Although many of the infections are asymptomatic, it can produce a self-limited but debilitating clinical presentation characterized by high fever, headache, retroorbital pain, myalgia, arthralgia, nausea, vomiting, lymphadenopathy and rash. The major risk of DENV infection is for those patients who develop dengue-hemorrhagic fever (DHF) which can be death threatening (3). DHF has three phases: febrile, critical and recovery. In the critical phase the increase in capillary permeability leads to plasma leakage and hypovolemic shock with multiorgan failure, metabolic acidosis, disseminated intravascular coagulation and hemorrhage (4). Some critical patients can develop hepatitis, encephalitis, myocarditis, and severe hemorrhage without plasma leakage. In these cases, intravenous rehydration treatment can reduce mortality from 20% to 1% (3).

Four DENV serotypes (1–4) exist, sharing between 60%-70% of their coding sequence (5). DENV pathogenicity in the human host can be partially explained by differences in viral virulence due to genotype and serotype (6). For instance, the Asian genotype of DENV-2 produces a more severe disease than the American genotype (7, 8). Host factors are also implicated in the severity of the disease, including the humoral immune response. The immune response against DENV differs between serotypes, a serotypic-specific or homotypic response is produced against the infecting serotype while a cross-reacting or heterotypic response is generated against other serotypes (9). A heterotypic immune response provides protection for an estimated period of six months to three years while a homotypic immune response should give a lifetime protection (10). However, once a cross-reacting immune response cannot protect the host anymore, it can contribute to the immunopathogenesis of the disease by exacerbating inflammation through a cytokine storm or immunopotentiation (ADE) (11). The overproduction of cytokines produces endothelial cell damage increasing vascular permeability and plasma leakage characteristic of DHF (12). Complement activation and the production of a temporal autoimmune response may also occur (13, 14).

Both the cellular and humoral heterotypic immune response may induce immunopathogenesis. Cross-reacting cytotoxic T cells are ineffective at controlling the infection and increase the production of cytokines (15). Antibody-dependent enhancement (ADE) of infection occurs when IgG antibodies bind the viral particles but are uncapable to neutralize them and instead, form immune complexes that bind to the Fcγ receptors (FcγR) on immune cells, favoring viral infection of these cells followed by uncontrolled immune cell activation (16). Antibody specificity determines the risk of developing ADE. Antibodies targeting the I- and II- domain of the envelope (E) viral glycoprotein are highly serotype cross-reactive and associated with ADE (11, 17). The tridimensional disposition of the epitopes and antibody concentration also has an impact on the development of ADE (18). Linear epitopes and neutralizing antibodies at low concentrations can favor ADE (19, 20). Thus, serotype-specific, and cross-reactive antibodies may produce ADE depending on their concentration (18).

Fcγ receptors (FcγRs) belong to the immunoglobulin superfamily and are expressed on the surface of various immune cells, including monocytes, macrophages, neutrophils, and NK cells. The three main classes—FcγRI (CD64), FcγRII (CD32), and FcγRIII (CD16)—differ in structure, cellular distribution, affinity for IgG subclasses, and the signaling pathways they activate (21). In the context of DENV infection, FcγRs play a dual role: they can mediate protective immune clearance or contribute to ADE, depending on the antibody characteristics and the receptor involved. Notably, FcγRIIIa (CD16a), expressed primarily on NK cells and some myeloid populations, has been implicated in both beneficial effector functions such as antibody dependent cell-mediated cytoxicity (ADCC) and potentially in facilitating ADE under certain conditions (22, 23). Despite its relevance, the dynamics of FcγRIIIa activation during acute dengue infection remain poorly understood. In this study, we aim to characterize the FcγRIIIa-activating antibody profile in individuals with acute dengue infection, evaluate how it relates to other antibody effector functions and compare it to the profile found in convalescent patients.

## 2 Materials and methods

### 2.1 Serum samples

Two sets of serum samples were analyzed. A first set consisted of seven anonymous convalescent serum samples (S) collected in Golfito and Puntarenas, which represented DENV hyperendemic regions in Costa Rica, for a previous sero-epidemiological study during 2005-2006 (24). The second set of samples were collected from seven acute dengue adult patients with follow-up serial sample collections (**Table 1**). All sera were collected from non-severe dengue cases. Previous exposure to DENV infection was assessed with IgG detection in the acute sample with a commercial ELISA. Individuals with IgG antibodies against DENV during acute infection were categorized as non-primary infection (NP) and those where antibodies were not detected were classified as primary infection (P). Ethical approval for the use of human samples was given to the project B7360 in the resolution VI-3178–2017 by the Scientific Ethical Committee from the Vice rectory of Research of the University of Costa Rica.

**Table 1:** List of samples from individuals with a confirmed acute infection and with sequential sample collection at different time points (T1-T4), including the number of days post-symptom onset for sample collection.

ID	Infecting DENV Serotype	Days post-symptoms onset			
		Sample 1	Sample 2	Sample 3	Sample 4
		(T1)	(T2)	(T3)	(T4)
P1	DENV-3	3	14	82	–
P2	DENV-3	2	13	81	–
NP1	DENV-1	2	17	–	–
NP2	DENV-1	4	21	–	–
NP3	DENV-1	4	33	127	–
NP4	DENV-1	6	36	116	–
NP5	DENV-1	2	11	48	1719

NP, non-primary infection; P, primary infection.

### 2.2 Anti-DENV IgG and IgM detection

To detect IgG and IgM antibodies against DENV, two highly sensitive commercial ELISA kits were used (26): the Human Dengue IgG ELISA Test Kit (Diagnostic Automation, Cortez Diagnostics Inc., CA, USA) with 94.7% sensitivity and 97.4% specificity, and the Human Dengue IgM ELISA Test Kit (Diagnostic Automation, Cortez Diagnostics Inc., CA, USA) with 97.8% sensitivity and 93.5% specificity. Both assays were performed following the manufacturer’s protocol. Optical density (OD) values were measured after a 25-minute reading at 450 nm and 630 nm using the Epoch spectrophotometer (BioTek, Vermont, USA).

### 2.3 Molecular detection and serotyping of DENV

Viral RNA was extracted from 200 μl of serum or urine using the MagNA Pure LC RNA Isolation Kit I (Roche, Basel, Switzerland) according to the manufacturer’s instructions, using the MagNA Pure LC 2.0 extraction system (Roche, Basel, Switzerland). Detection and confirmation of DENV, ZIKV, and CHIKV were conducted on RNA samples using real-time reverse transcription PCR (RT-PCR) with Modular Diagnostic Kits for Dengue, Zika, and Chikungunya viruses, along with Multiplex RNA Master Mix on the LightCycler II (Roche, Basel, Switzerland), following the manufacturer protocol. Dengue serotyping was carried out following the protocol described by Lanciotti et al., using specific serotype controls (25).

### 2.4 Viral strains and cell lines

Dengue virus prototype strains all grown in the C6/36 cell line (ATCC^®^ CRL-1660™ RRID: CVCLZ230), donated by the *Pedro Kourí Institute* in Cuba, were used in the K562 (ATCC^®^ CCL-243™ RRID: CVCL0004) immune enhancement and FcγRIIIA–CD3ζ activation assays (26). The strains were DENV-1 Angola (12 passages), DENV-2 Jamaica (19 passages), DENV-3 Nicaragua (13 passages) and DENV-4 Dominica (16 passages).

For neutralization assays, chimeric viruses (ChimeriVax – DENV1, DENV2, DENV3 and DENV4) produced by Sanofi Pasteur and grown in Vero cells (ATCC^®^ CCL-81™ RRID: CVCL0059) were used (27). These viruses are based on the yellow fever 17D vaccine backbone and express only the prM and E genes of each DENV serotype, thereby assessing the neutralizing activity of antibodies directed against the major structural antigens involved in viral entry. Using this approach restricts the readout to neutralization-relevant epitopes, thereby minimizing contributions from other viral proteins. These viruses were donated by Sanofi Pasteur through the CDC Arbovirus Reference Collection under a material transfer agreement (MTA).

### 2.5 Reporter cell BW: FcγRIII-ζ assay

The assay used to evaluate individual antibody-dependent activation of FcγRIII (CD16) involved co-culturing antigen-bearing cells with BW5147 reporter cells that stably express chimeric FcγRIII-ζ chain receptors. These receptors trigger mouse IL-2 production upon receptor crosslinking by immune-complexed IgG, provided the opsonizing IgG is recognized by specific FcγR (26). This assay was standardized before in Corrales-Aguilar et al. (26). Briefly, to assess antibody-dependent activation of BW: FcγRIII-ζ reporter transfectants, Vero cells where infected with 0.1 multiplicity of infection (MOI) of each DENV serotype for a 72-hour period, then virus was inactivated by UV-light. After inactivation, mock-infected and virus-infected cells were incubated with serial two-fold dilutions of human sera in D-MEM (Sigma-Aldrich, MO, USA). containing 10% (v/v) FCS (Thermo Fisher Scientific, MA, EE.UU.) for 30 minutes at 37 °C in a 5% CO_2_ atmosphere. Non-bound IgG was removed by washing the cells three times with D-MEM containing 10% (v/v) FCS before co-culturing them with 100–000 BW: FcγRIII-ζ reporter cells per well for 16 to 24 hours at 37 °C in a 5% CO_2_ atmosphere in RPMI medium (Thermo Fisher Scientific, MA, EE.UU.) supplemented with 10% (v/v) FCS. Unless otherwise noted, experiments were conducted in triplicate with a MOI of 0.1. After the 16 to 24-hour co-cultivation, supernatants were diluted 1:2 in ELISA sample buffer (PBS with 10% [v/v] FCS and 0.1% [v/v] Tween-20). Mouse IL-2 levels were then measured by ELISA using the capture antibody JES6-1A12 and the biotinylated detection antibody JES6-5H4 (BD Pharmingen™, Erembodegem, Belgium. RRID: AB2067783 and RRID: AB2621654 respectively) following the manufacturer instructions. The cutoff point for result interpretation was calculated by adding the mean of mIL-2 production in virus-free (or mock) cells and three standard deviations. Values above this cutoff point were considered positive. The magnitude of IL-2 production was interpreted as an indicator of the strength of receptor engagement by IgG–virus immune complexes. Higher IL-2 values reflect more efficient crosslinking of FcγRIIIA (26, 28).

### 2.6 Focus reduction neutralization test

For the FRNT assay, ChimeriVax strains (YFV-DENV1, 2, 3, and 4), validated for viral neutralization studies (29), were used. A focus-reduction microneutralization assay (FRNT) was performed in flat-bottom 96-well plates (30). Serial two-fold dilutions of sera, starting at 1:40, were incubated for 1 hour at 37°C with viral stocks, adjusted to yield 30–200 foci per well in at least four wells. The mixture was then inoculated (50 μL/well) into confluent Vero cell monolayers and incubated for an additional hour to allow viral adsorption. The adsorption medium was replaced by 100 μL of 1.5% carboxymethylcellulose overlay medium to restrict infection. DENV-1, DENV-2, and DENV-3 were incubated at 37°C for 48 hours, while DENV-4 was incubated for 24 hours. Post-incubation, the overlay medium was removed, wells were washed with PBS (Thermo Fisher Scientific, MA, EE.UU.) and fixed with 100 μL of cold methanol per well. Plates were stored at −20°C for at least 24 hours. For focus visualization, immunostaining was performed using an anti-flavivirus group monoclonal antibody 4G2 (GeneTex, CA, USA. RRID: AB3074294) (1:600 dilution) followed by a secondary anti-mouse IgG antibody conjugated with peroxidase (1:600 dilution). The signal was developed using 3-amino-9-ethylcarbazole (AEC) substrate, incubated for 30 minutes at room temperature in darkness. Foci were imaged using a stereoscope and manually counted with ImageJ software (RRID: SCR_003070). FRNT50 was determined in Prism 10 (GraphPad, San Diego, CA, USA) by nonlinear regression, identifying the dilution that reduced foci by 50% (FRNT50). High FRNT50 values indicate stronger neutralizing capacity against the tested DENV serotype.

### 2.7 Antibody dependent enhancement test

This study used the semi-adherent K562 cell line, which constitutively expresses FcγRIIa (31), based on a monolayer methodology (32). Plates were coated with fibronectin and 30–000 cells per well were added. Serial dilutions of test sera were mixed with DENV serotypes at a MOI of 0.5 (DENV4) to 0.1 (other serotypes) and incubated at 37 °C for 24 (DENV-4) to 48 (other serotypes) hours. Post-incubation, cells were fixed, immunostained with the 4G2 antibody and secondary anti-mouse peroxidase-conjugated antibodies and stained with AEC to visualize infected cells as described before. Infected cells, identified by a precipitated brown color, were observed under light microscopy, and the number of infected cells per 40X field was quantified using ImageJ software. The percentage of infection for all serial dilutions was plotted, and the level of immunopotentiation was determined based on the width of the curve. Samples that exhibited broad curves against more than one DENV serotype were considered to have a high level of immunopotentiation as defined in other studies (18). The magnitude of enhancement was interpreted based on the breadth and height of the curve: narrow, low curves were considered low enhancement, whereas broad curves with high percentages of infection across multiple dilutions indicated strong enhancement potential.

### 2.8 Statistical analysis

All assays were performed in triplicate unless otherwise indicated. Data are shown as individual values or as mean ± standard deviation (SD). For the FcγRIIIA activation assay, the cutoff for a positive response was defined as the mean IL-2 production of mock-infected cells plus three standard deviations. Neutralization titers (FRNT50) were determined by nonlinear regression analysis using GraphPad Prism 10 (GraphPad Software, San Diego, CA, USA). No formal hypothesis testing was performed due to the small sample size; instead, results are presented descriptively to illustrate individual antibody profiles over time.

## 3 Results

### 3.1 Serological and functional characterization of samples

Serum samples were classified into two main groups based on clinical and serological criteria: past infections and acute infections. The past infection group (S) consisted of asymptomatic individuals with serological evidence of prior DENV exposure, while the acute infection group included laboratory-confirmed cases of active dengue virus infection. Acute-phase samples were further subdivided into primary (P) and non-primary infections (NP), based on the presence or absence of anti-DENV IgG within the first seven days following symptom onset. The detection of IgG at this early stage was used as a proxy to distinguish primary infections from those that were likely secondary or beyond. Due to limitations in discriminating between secondary and tertiary or quaternary responses, all early IgG-positive acute cases were conservatively grouped as non-primary (NP) infections.

**Figure 1:**
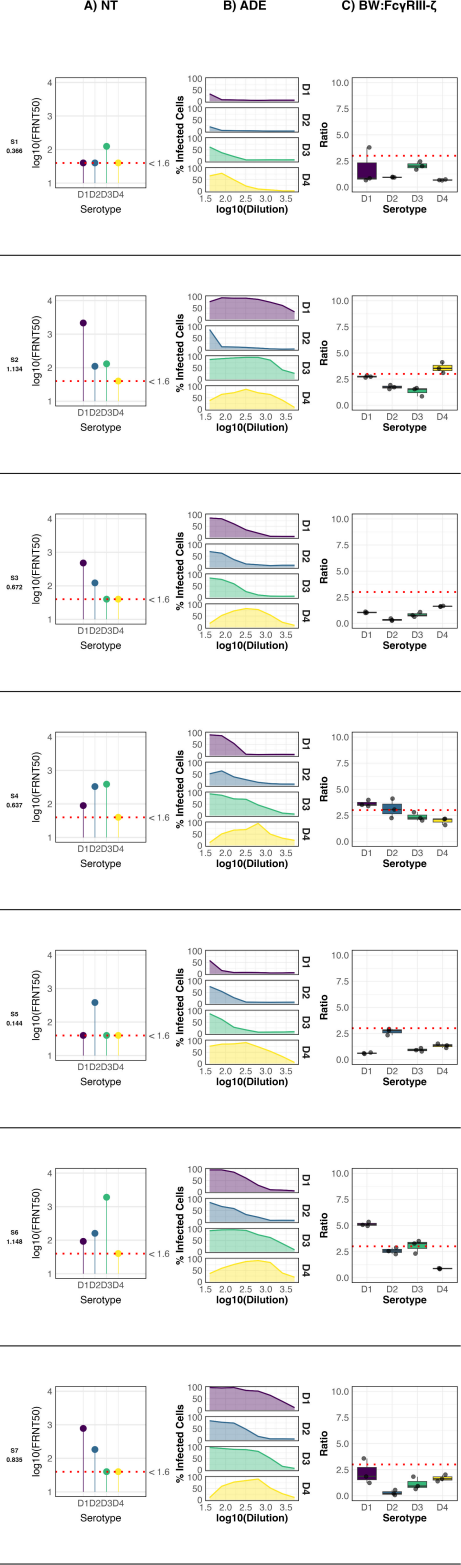
Anti-DENV antibody profile of seven participants from a serosurvey. The ELISA OD values for IgG detection are indicated below each participant code. NT: Neutralization profile; ADE: Antibody-dependent enhancement profile; BW: CD16: FcγRIIIA activation profile. For FcγRIIIA activation, data points represent the mean of three independent experiments ± standard deviation (SD), and the cutoff for a positive response (dotted line) was defined as the mean IL-2 production of mock-infected cells plus three standard deviations. Neutralization titers (FRNT50) were determined by nonlinear regression analysis. Negative controls included mock-infected cells (for FcγRIIIA assay) and seronegative human sera (for ELISA, ADE, and FRNT assays).

The samples from past infections presented highly diverse profiles depending on IgG antibody concentration measured as OD values. Samples with anti-DENV IgG optical density (OD) values below 0.500 displayed a monotypic neutralization profile, showing serotype-specific activity restricted to either DENV-3 (**Figure 1**, Sample 1 (S1)) or DENV-2 (**Figure 1**, Sample 5 (S5)).These specimens exhibited minimal ADE activity, revealed by the short breath of the curves against the four serotypes, and failed to induce significant activation of the FcγRIIIA–CD3ζ receptor, suggesting limited effector function in this group.

In contrast, samples with intermediate anti-DENV ELISA OD values (0.5–1.0) exhibited broader serotype recognition, neutralizing two (**Figure 1**, Samples 3, 7 (S3, S7)) or three (**Figure 1**, Sample 4 (S4)) DENV serotypes. Moderate ADE activity was observed across these samples. Notably, FcγRIIIA–CD3ζ activation was detected exclusively in S4. Interestingly, despite having the highest neutralizing titer against DENV-3, the strongest receptor activation in S4 was induced by DENV-1, highlighting a potential uncoupling between neutralization capacity and Fc-mediated effector activation.

Only two samples exhibited high anti-DENV IgG OD values (>1.0). Both (**Figure 1**, Samples 2, 6 (S2, S6)) neutralized three serotypes and displayed the highest levels of ADE and FcγRIIIA–CD3ζ activation among all specimens analyzed from the past infection cohort. S2 showed peak FcγRIIIA activation in response to DENV-4, with neutralization strongest against DENV-1. In contrast, in S6 the strongest receptor activation occurred in response to DENV-1, while the highest neutralization titer targeted DENV-3. These findings underscore the complex relationships among antibody specificity, enhancement potential, and Fc-mediated effector functions following natural DENV exposure.

### 3.2 Longitudinal analysis of serum samples from acute DENV infections

**Figure 2:**
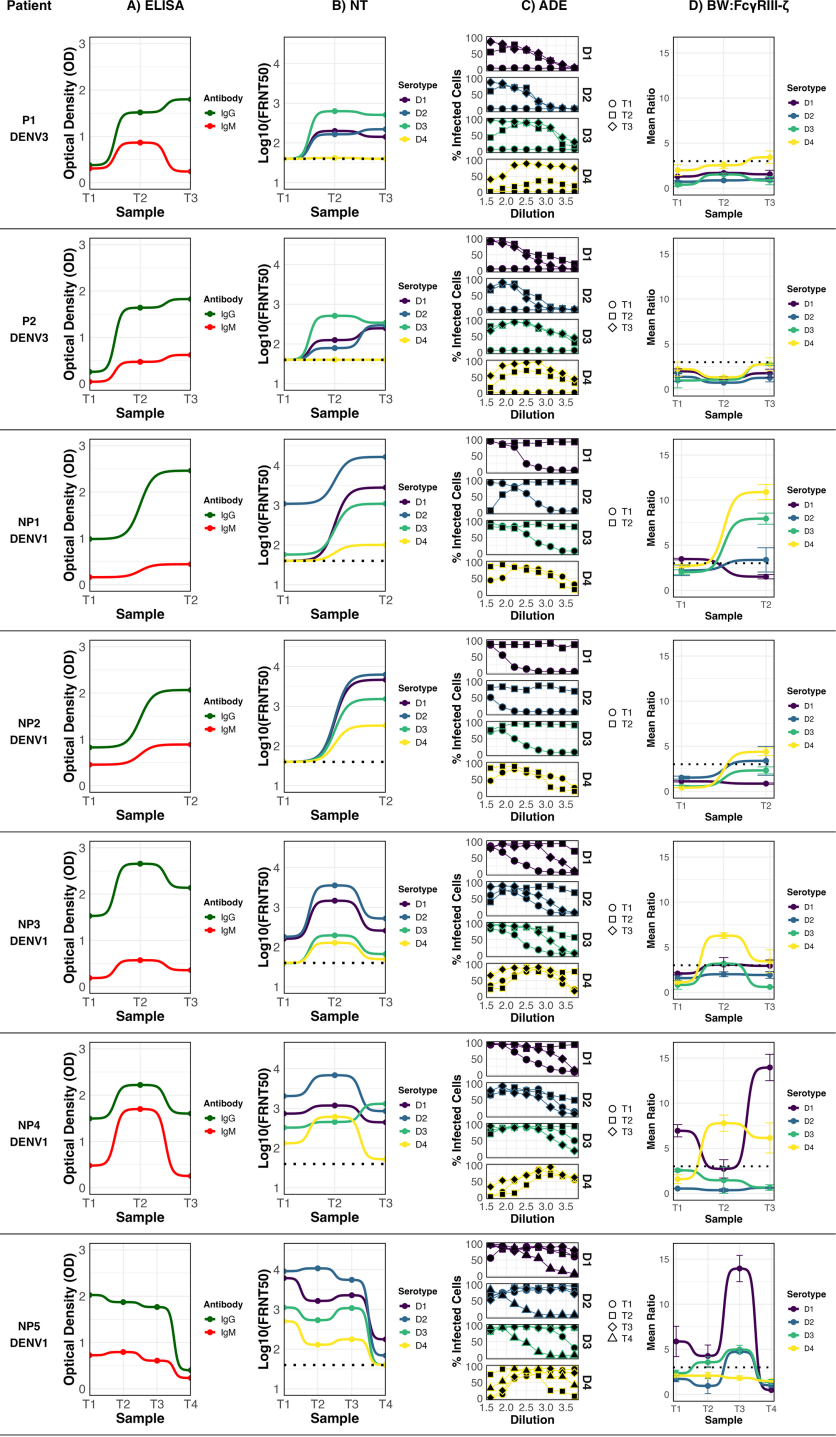
Anti-DENV antibody profile of seven dengue patients with sequential samples collected at different time points (T1–T4) post-symptom onset. Infecting serotype is indicated below each patient. ELISA: anti-DENV IgG and IgM profile; NT: Neutralization profile; ADE: Antibody-dependent enhancement profile; BW: CD16: FcγRIIIA activation profile. For FcγRIIIA activation, data points represent the mean of three independent experiments ± standard deviation (SD), and the cutoff for a positive response (dotted line) was defined as the mean IL-2 production of mock-infected cells plus three standard deviations. Neutralization titers (FRNT50) were determined by nonlinear regression analysis. Negative controls included mock-infected cells (for FcγRIIIA assay) and seronegative human sera (for ELISA, ADE, and FRNT assays).

In the longitudinal study, the values for all antibody characterization assays for each patient were plotted across all collected samples (T1-T4) (**Figure 2**). In primary DENV infections (**Figure 2**, P1 and P2), the immune response followed classical kinetics, marked by the induction of anti-DENV IgG and a progressive increase in functional activity. Neutralization peaked at T3 timepoint, with strong titers against the infecting serotype (DENV-3). ADE activity rose during the T2 timepoints, with moderate levels persisting in the T3 subacute phase. FcγRIIIA–CD3ζ activation was largely absent, except for a minimal, above-threshold response to DENV-4 in P2 at T3.

In non-primary infections (NP), antibody dynamics were more heterogeneous. In all cases, the infecting serotype was DENV-1. Patient NP2 showed detectable IgG during the acute phase, but without measurable neutralizing activity. By T2, neutralization peaked against DENV-2, and ADE activity increased notably. A low but detectable FcγRIIIA–CD3ζ activation signal was recorded in T3 against DENV-4 (**Figure 2**, NP2). For NP3, all measured antibody activities—including neutralization, ADE, and FcγRIIIA–CD3ζ activation—peaked at T2 and declined by T3 timepoint. Neutralizing responses were strongest against DENV-2 across all timepoints, followed by DENV-1, suggesting that DENV-2 was likely the priming serotype. FcγRIIIA activation in this case was restricted to DENV-4, which peaked at T2 and decrease by T3 (**Figure 2**, NP3).

NP5 was the only case with a fourth sample collected nearly five years post-infection (**Figure 2**, NP5). The acute-phase sample (T1) showed the highest IgG OD value among all evaluated samples, along with the strongest neutralizing response against DENV-2, followed by DENV-1, supporting DENV-2 as the primary infecting serotype. FcγRIIIA–CD3ζ activation was significant for DENV-1, DENV-2, and DENV-3, with DENV-1 showing the highest signal from T1. An elevated enhancing activity is seen for all serotypes in all time points. It should be noted that in this case the acute sample had a broad neutralizing activity, recognizing all four serotypes. Although antibody function remained relatively high through T3, all profiles declined markedly by T4.

Patient NP4 showed persistently high IgG OD values and a broad neutralization activity across all timepoints, being the strongest against DENV-2, suggesting this serotype as the primary exposure. ADE activity increased over time, and FcγRIIIA–CD3ζ activation was pronounced against DENV-1 from the acute phase through T3. A secondary, though significant, activation signal was also observed for DENV-4 (**Figure 2**, NP4). In the case of NP1, the neutralization profile also pointed to DENV-2 as the primary infecting serotype. Enhancing activity was initially low but increased by T2. FcγRIIIA–CD3ζ activation was undetectable in the acute phase, but increased significantly in the subacute sample, particularly in response to DENV-4, DENV-3, and DENV-2.

Collectively, these findings highlight the dynamic and individualized nature of DENV-specific antibody responses following natural infection. Primary infections showed a more predictable trajectory of rising neutralization and ADE activity, with minimal detection of FcγRIIIA activation. In contrast, non-primary infections were characterized by broader serotype recognition, variable neutralization targets, and a more prominent engagement of FcγRIIIA-mediated triggering. Notably, patients NP4 and NP5—who exhibited the broadest neutralization profiles—were also the only individuals with the detectable FcγRIIIA activation against the infecting serotype (DENV-1) with peaks at relatively late time-points (T3) after symptom onset.

## 4 Discussion

In this pilot study, we determined for the first time distinct antibody effector functions and profiles by ELISA, FRNT, ADE test and FcγRIIIA activation assay across different immunological contexts of DENV 1–4 infection. Notably, in most cases, FcγRIIIA–CD3ζ activation did not consistently correlate with neutralization profiles, one explanation may be that the epitopes driving neutralization differ from those responsible for Fc-mediated functions (28). Neutralization is typically mediated by antibodies targeting structurally critical regions on the virion, such as quaternary epitopes recognizing multiple envelope (E) protein subunits or serotype-specific sites on the E protein domain III (33). By contrast, robust FcγRIIIA activation often arises from highly cross-reactive IgG antibodies against conserved epitopes that confer little DENV neutralization​. Notably, many human anti-DENV antibodies dominantly target the precursor membrane (prM) protein and the conserved fusion-loop of E domain II; these antibodies are broadly cross-reactive among serotypes yet poorly neutralizing, even at high concentrations, and can still efficiently opsonize infected cells and virions, triggering FcγRIIIA (17, 33). Indeed, the FcγRIIIA activation assay of this study utilized DENV-infected Vero cells that display both E and uncleaved prM on their surface, providing abundant targets for Fc binding in comparison to neutralization assay​ (34). In summary, the antigenic determinants of neutralization versus FcγRIIIA-mediated effector function only partially overlap, leading to an uncoupling dissection of these profiles in many samples.

Past infection data likely reflect a range of diverse time points post DENV infection (**Figure 1**). Samples with broader serotype reactivity and enhanced Fc-mediated function are consistent with non-primary infections or specimens taken within two years after exposure, when cross-reactive antibodies remain elevated (35). Longitudinal analysis of acute cases provides a clearer view on the kinetics of the humoral response and its associated effector functions. Individuals with secondary or multiple infections exhibited notably stronger FcγRIIIA–CD3ζ activation compared to primary cases. This increased activity reflects not only higher antibody titers but also qualitative differences in the IgG response, possibly due to subclass distribution and Fcγ N297 glycosylation pattern as demonstrated in COVID-19 patients (36–38). DENV infection predominantly induces IgG1 and IgG3, both capable of engaging FcγRIIIA. IgG3 is short-lived and more potently neutralizing, while IgG1 is longer-lasting and subject to glycan modification (22). It has been described that afucosylation of IgG1 is more prominent in dengue secondary infections, and that elevated levels of afucosylated anti-E IgG1 are present early on severe dengue (22). Afucosylation significantly enhances FcγRIIIA binding (22) which may explain the difference observed between P and NP individuals.

Analysis of past infection samples revealed a consistent association between the breadth of serotype recognition by neutralization and the magnitude of FcγRIIIA-mediated effector activity. In acute infections, broadly neutralizing sera, typically from those with non-primary infection, tended to activate FcγRIIIA across multiple serotypes more robustly than narrow, type-specific sera. A broader neutralization profile implies a more extensive distribution of IgG bound to diverse epitopes on the virion surface or the infected cells membrane, thereby increasing the valency, defined as the multivalent engagement of antibodies with multiple epitopes, and the density of immune complexes (22). This configuration enhances the odds of cross-linking of FcγRIIIA on effector cells, a prerequisite for efficient receptor signaling (39). This finding is consistent with the concept that a minimum concentration and opsonization density of IgG must be achieved to overcome the activation threshold of FcγRIIIA. Prior studies of dengue immunity have noted that intermediate antibody levels can exacerbate infection (via ADE), but sufficiently high antibody levels confer protection (40, 41). Analogously, only the samples with high IgG binding levels were potent in FcγRIIIA triggering, whereas those with modest titers did not (23, 42). Thus, a higher abundance and breadth of antibodies likely ensures that FcγRIIIA is engaged in antiviral effector functions rather than in enhancing pathways.

The longitudinal FcγRIIIA activation profiles observed in individuals NP5 and NP4 provide valuable insight into the dynamics of Fc-mediated antibody responses during acute dengue infection. In both cases, a marked FcγRIIIA/CD16 activation signal was detected in response to the infecting serotype (DENV-1) during the acute phase, indicating the presence of FcγRIIIA-activating IgG early in infection. Interestingly, both individuals exhibited a transitory decline in activation at the T2 timepoint, followed by a peak in T3. This transient reduction may reflect *in vivo* engagement of FcγRIIIA-expressing effector cells, such as natural killer (NK) cells or monocytes, by IgG-virus immune complexes, leading to ADCC or phagocytosis and temporary clearance of activating antibodies in immune complexes (22, 23, 42, 43). The increased CD16 activation signal observed in T3 may be due to clonal expansion against the epitopes recognized in the acute infection (44). Notably, while both individuals shared similar FcγRIIIA activation kinetics against the infecting serotype, they differed in their ADE profile: NP5 displayed high ADE in acute sample, while NP4 did not. This immune assessment enabled the distinction between FcγRIIIA-activating antibody profiles with low enhancing potential and those with strong enhancing activity.

Our study focused exclusively on FcγRIIIA activation profile, which does not capture the full range of FcγR-mediated effector mechanisms. Furthermore, FcγR polymorphisms such as FcγRIIA-H131R and FcγRIIIA-V158F, which affect the affinity of Fcγ receptors for IgG subclasses, have been associated with increased susceptibility and protection against severe dengue, respectively (45, 46). Therefore, a broader approach incorporating additional FcγRs, and their key polymorphic variants, along with FcγR reporter cell assay settings selective for certain ligands including soluble multimeric immune complexes and C reactive Protein isoforms (47, 48), should be undertaken to evaluate the full effector potential of dengue-specific antibodies and to identify thresholds that help define the spectrum of clinical outcomes from DENV infection. The hyperendemic setting in Costa Rica, where multiple flaviviruses co-circulate, highlights the need for a broader viral panel to better interpret antibody profiles. This would allow for the inclusion of both severe and non-severe patients (2). Increasing the number of patients, outcomes of DENV-disease, and timepoints during the early acute and convalescent phases would provide a more detailed understanding of how FcγR activation evolves. This would also help to clarify its complex role in the dual nature of the humoral response in dengue infection.

Recent studies highlight the dual impact of FcγRIIIA interactions: afucosylated IgG1 enhancing FcγRIIIA binding has been linked to severe dengue (23, 42, 49, 50), dengue immune complexes can activate NK cells and suppress ADE (51), and stronger FcγRIIIA-driven effector functions, including NK activation, associate with protection from symptomatic infection (22). While NK cell–based assays are highly informative to evaluate the protective role of CD16-activating antibodies, our reporter system allows the measurement of the broader fraction of antibodies capable of engaging FcγRIIIA, including those that may also contribute to immunopathogenic outcomes, since FcγRIIIA expression is not restricted to NK cells but includes monocytes implicated in infection and inflammation (52). This distinction provides a complementary view, revealing potentially different functional profiles of dengue antibodies. Additionally, Kao et al. recently revealed that CD8 T cells, which typically do not express Fcγ receptors, can specifically induce the activating FcγRIIIa receptor in response to viral infections like COVID-19 and dengue (53). While FcγRIIIa expression closely follows the immune response timeline, its activation alone does not trigger CD8 T cell function; however, it synergizes with T cell receptor (TCR) stimulation to enhance activation (53). These findings uncover a novel costimulatory role for FcγRIIIa, showing how virus-induced antibodies can modulate CD8 T cell responses. By providing a scalable and reproducible way to measure FcγRIIIA engagement beyond natural killer and CD8 T cell functions, our assay offers a novel framework to characterize the balance between protective and pathogenic antibody responses.

Taken together, our data shows that neutralization and FcγRIIIA-mediated antibody functions against Dengue viruses are often uncoupled which has already been observed with other viral infections before (28). Furthermore, the different epitopes involved in each process may lead to distinct antibody functional profiles. Cross-reactive antibodies (e.g., anti-prM, fusion-loop) may not neutralize dengue virus effectively but still trigger immune effector mechanisms via Fc receptors. To better understand how antibody effector mechanisms and Fc-mediated immunity influence dengue outcomes, different FcγRs and their polymorphisms, distinct immune complex forms, more patients and defined timepoints of sampling should be studied. Our foremost rationale for using these tests will be to evaluate the functional quality of antibodies, especially cross-reactive ones, during different phases of dengue infection (acute and post-acute). This may help elucidate their dual role in both protection and immunopathogenesis, improving our understanding of disease progression and immune responses, and potentially guiding vaccine development by distinguishing between protective and pathogenic antibody profiles.

## Data Availability

The raw data supporting the conclusions of this article will be made available by the authors, without undue reservation.
